# Early and accurate detection of melanoma skin cancer using hybrid level set approach

**DOI:** 10.3389/fphys.2022.965630

**Published:** 2022-12-05

**Authors:** Mahmoud Ragab, Hani Choudhry, Mohammed W. Al-Rabia, Sami Saeed Binyamin, Ahmed A. Aldarmahi, Romany F. Mansour

**Affiliations:** ^1^ Information Technology Department, Faculty of Computing and Information Technology, King Abdulaziz University, Jeddah, Saudi Arabia; ^2^ Centre for Artificial Intelligence in Precision Medicines, King Abdulaziz University, Jeddah, Saudi Arabia; ^3^ Mathematics Department, Faculty of Science, Al-Azhar University, Nasr City, Egypt; ^4^ Biochemistry Department, Faculty of Science, King Abdulaziz University, Jeddah, Saudi Arabia; ^5^ Department of Medical Microbiology and Parasitology, Faculty of Medicine, King Abdulaziz University, Jeddah, Saudi Arabia; ^6^ Health Promotion Center, King Abdulaziz University, Jeddah, Saudi Arabia; ^7^ Computer and Information Technology Department, The Applied College, King Abdulaziz University, Jeddah, Saudi Arabia; ^8^ Basic Science Department, College of Science and Health Professions, King Saud Bin Abdulaziz University for Health Sciences, Jeddah, Saudi Arabia; ^9^ King Abdullah International Medical Research Center, Ministry of National Guard—Health Affairs, Jeddah, Saudi Arabia; ^10^ Department of Mathematics, Faculty of Science, New Valley University, El-Kharga, Egypt

**Keywords:** dermoscopy, skin lesions, cancer, level set, melanoma, lesion segmentation, computer aided design

## Abstract

Digital dermoscopy is used to identify cancer in skin lesions, and sun exposure is one of the leading causes of melanoma. It is crucial to distinguish between healthy skin and malignant lesions when using computerised lesion detection and classification. Lesion segmentation influences categorization accuracy and precision. This study introduces a novel way of classifying lesions. Hair filters, gel, bubbles, and specular reflection are all options. An improved levelling method is employed in an innovative method for detecting and removing cancerous hairs. The lesion is distinguished from the surrounding skin by the adaptive sigmoidal function; this function considers the severity of localised lesions. An improved technique for identifying a lesion from surrounding tissue is proposed in the article, followed by a classifier and available features that resulted in 94.40% accuracy and 93% success. According to research, the best method for selecting features and classifications can produce more accurate predictions before and during treatment. When the recommended strategy is put to the test using the Melanoma Skin Cancer Dataset, the recommended technique outperforms the alternative.

## 1 Introduction

Skin cancer can be successfully and affordably treated if detected early. Caucasians have an increased risk of developing malignant melanoma (MM). People with dark skin are more likely to develop MM. Early identification of skin cancer is critical for successful therapy. Melanoma, a kind of skin cancer, necessitates meticulous patient monitoring. Melanocytes are at the heart of the melanoma problem. Melanoma can develop in any part of the body, including the face, neck, arms, trunk, and legs. Visual examination is currently the most critical step in the diagnosis of melanomas. Within melanoma diagnosis, there are both malignant and noncancerous subtypes. Melanoma is a rare and possibly dangerous form of skin cancer. It has a coarse texture. Dermatoscopy, often known as dermoscopy, aids doctors in the detection of cancer. Digital dermoscopy provides more precision. Computer-aided design (CAD) systems use computerised images of malignancy to diagnose melanoma and its stages. For quick and precise cancer therapy, CAD technologies recognise a lesion’s illness signs. Because of the nature of this procedure, it is feasible to remove a skin lesion all the way down to the layer of fat beneath the dermis known as subcutaneous fat (the second layer of the skin). To ensure that no carcinogenic cells remain after the tumour has been treated, a piece of the surrounding healthy tissue will most likely need to be removed. When there is reason to think that a person has skin cancer, the likelihood that they will seek medical assistance for the illness increases. This is due to the person’s proclivity to seek treatment for the ailment.

An elliptical component is often eliminated since it is easier to heal the incision with stitches if the region excised is of uniform thickness. This section is roughly the size of a football in terms of overall dimensions. This is done in this manner because it makes repairing the tear much easier. Access to the fatty tissue contained within a lesion is likely to be required to entirely eradicate it. To ensure that the margins are clean, an extra margin surrounding the tumour that is three to 4 mm in diameter or bigger may need to be removed. It was sewn up several times to guarantee that the wound did not reopen. If the wound is large enough, traditional skin flaps or skin grafts can be used to replace the missing skin. Another viable alternative is to use artificial skin. Cryotherapy is a treatment method that involves exposing damaged tissue to temperatures below freezing in order to treat an injury or illness. This chemical may be beneficial for a variety of skin diseases, including warts, actinic keratoses, seborrheic keratoses, and molluscum contagiosum. It may be successful in this endeavour.

**FIGURE 1:**
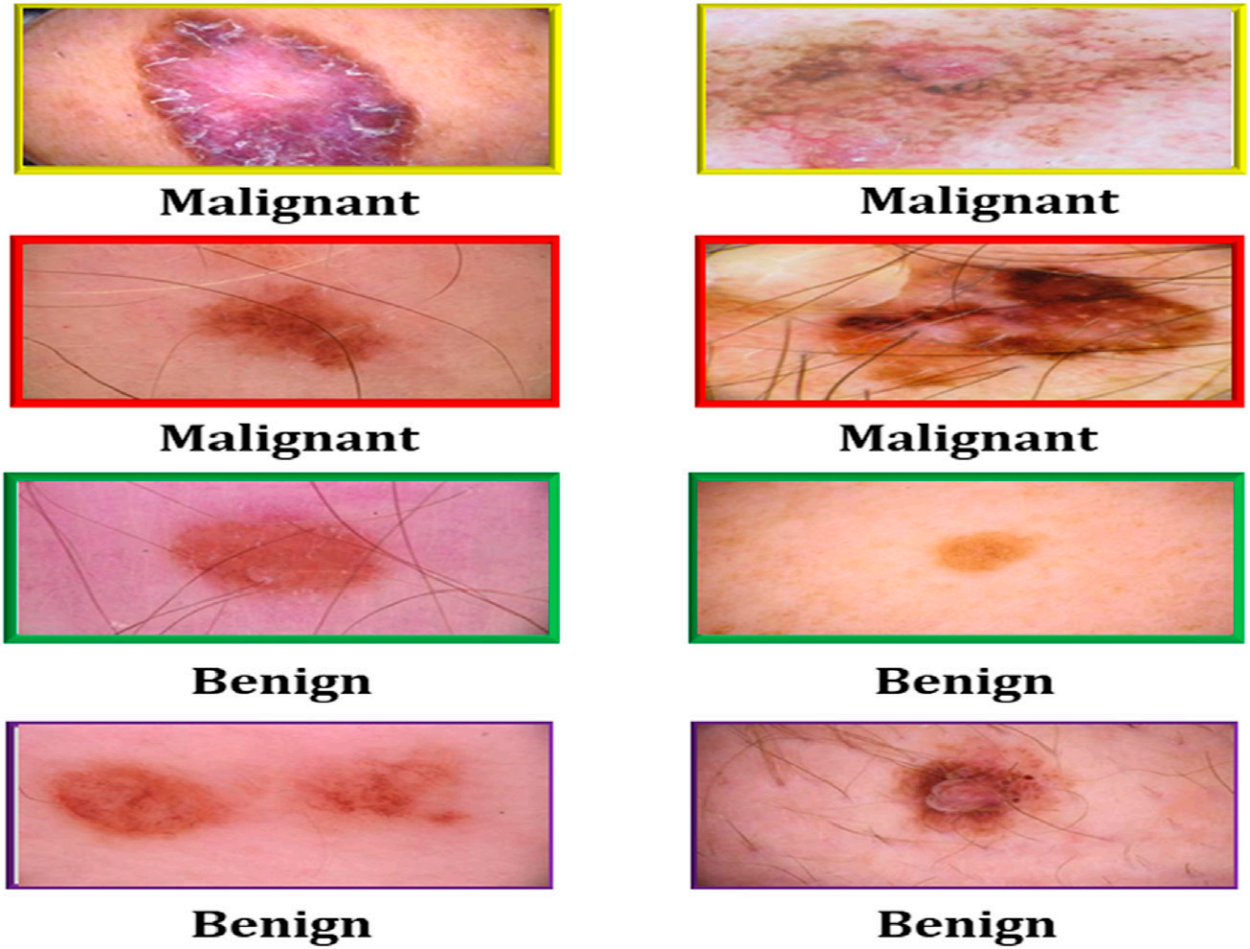
Malignant and benign skin cancer images of the melanoma skin cancer dataset (Melanoma Skin Cancer, 2022).

Cryotherapy can be administered using a cotton swab dipped in liquid nitrogen, a spray canister filled with the substance, or even a probe with liquid nitrogen running through it. These are only a few of the countless approaches available. Each of these strategies has its own set of advantages. Cryotherapy treatments can be administered using liquid nitrogen spray canisters in addition to traditional procedures. This strategy is just as effective as the others. The process takes less than a minute on average to complete. Automated melanoma detection consists of preprocessing, area extraction, postprocessing, and segmentation. Cleaning the data, integrating the data, reducing the data, and transforming the data are the four steps that comprise data preprocessing. These stages were created to make the process easier to manage. A digital image has been split. Non-lesion data is removed from images *via* image segmentation. Lesions’ shapes, sizes, skin types, and textures make segmentation difficult. Figure 1 shows images of malignant melanoma and benign dermoscopy; these artefacts may have an influence on feature computation and skin cancer classification. The lesion is separated after artefacts are removed. Following segmentation, we find and quantify melanoma subtypes.

Melanoma cells can originate in the tumour, but they can also move through the lymphatic and vascular systems to other organs and tissues, where they can form new tumors. Melanoma cells can develop from bloodstream cells. There is usually no cure for melanoma once it has migrated to other parts of the body from the spot where it was discovered as a tumour. Melanoma is the deadliest form of skin cancer. Melanoma can appear anywhere on the body. Because they have been exposed to the sun, they are more likely to grow on a person’s back, legs, arms, and face. Melanomas can appear anywhere on the body, even in regions that are rarely exposed to sunlight, such as the soles of your feet, the gaps between your fingernails, and your palms. These traits are used by melanoma classifiers (Khalid and Razzaq, 2012; Khalid and Arshad, 2013; Waheed et al., 2015), and this research focuses on the preprocessing and segmentation of dermoscopic melanoma (Khan and Khalid, 2015). It is easy to tell the difference between healthy and cancerous tissue, and skin cancer has gotten a lot worse in the last few decades (Abbas et al., 2012).

It is difficult to train neural networks for automatic recognition of pigmented skin lesions since the present collection of dermatoscopic images is both small and lacking in variation. This is one of the factors that makes this task difficult. This makes the training process more challenging. The authors acquired a collection of dermoscopic photographs of individuals from various population segments. These images were taken using a variety of methods, and they were then stored. The resulting dataset contains 10,015 dermatoscopic images, each of which could be used as a training set for academic machine learning applications. ImageJ, an application, was used to produce the dataset. Actinic keratoses, intraepithelial carcinoma (Bowen’s disease), basal cell carcinoma, and benign keratosis-like lesions (solar lentigines, seborrheic keratoses, and lichen-planus-like keratoses, or bkl) are all examples of pigmented lesion diagnosis. Melanomas begin in melanocytes, which produce melanin. Dark melanin causes melanin-rich skin to darken when exposed to sunlight. Melanocytes in the epidermis and dermis of the skin are responsible for skin color. Melanocytes are skin pigmentation cells; local surgery may be an option when the tumor’s diameter is less than 1 mm. We picked a tumour image because there is no blood test for melanoma, and cancer is difficult to diagnose because of its capacity to spread throughout the body. The dermis is the skin’s most visible layer, and ultraviolet radiation causes skin mutagenesis. It can affect skin that is pale or dark, freckled or naturally freckled, or that has a familial history of freckling. Melanoma is more likely to develop in those who have freckles. Even with advances in medical research, it can be difficult to diagnose amelanotic melanoma in a patient. If a new lesion or a change in the appearance of an existing lesion is discovered during a skin check, the patient should be instructed to return as soon as possible for a follow-up examination. As part of a skin cancer screening, both the patient and the healthcare practitioner can undergo a visual self-exam. If a doctor or nurse notices anything strange about a patient’s moles, birthmarks, or other pigmented patches on their skin, they will extensively inspect them. This is possible since the anomaly could be an indication of a more serious disease. This procedure entails checking the skin for signs of cancer.

We devised a segmentation method to remove skin lesions while also accounting for gel, bubbles, hairs, arteries, and fluctuations in the contrast of the underlying skin image. This strategy was devised entirely by the authors of this study. In this work, we present a unique strategy for dealing with hairs and veins quickly, obtaining more information regarding brightness, and increasing the contrast between pixels on the skin and pixels on the lesion. This aids in the segmentation of the lesion, and the application of directed wavelet filters allows for the amplification or identification of hairs and small vessels. We can solve difficulties swiftly because an inpainting process is used to fill in the hair and vessel pixels. It is feasible to correct uneven brightness by using non-uniform light in combination with luminance equalisation.

**FIGURE 2:**
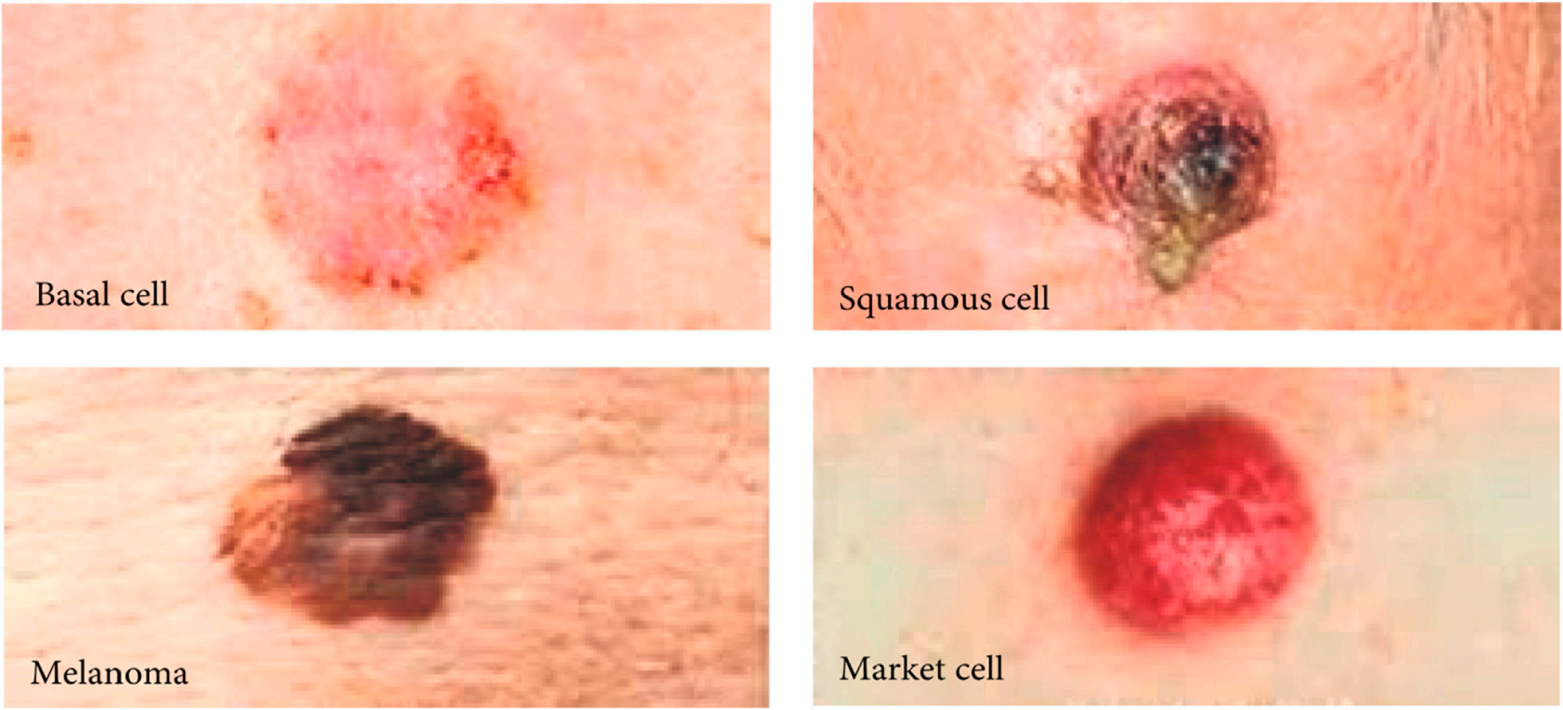
Types of skin cancer cell.

The contrast between the skin and the lesions may be reduced by sigmoidal functions. This approach employs cuts that are unique to each image. Thresholding and other morphological methods are used to distinguish the lesion from the skin that surrounds it. Images acquired from the Melanoma Skin Cancer Dataset are utilised to evaluate the procedure. The colour and texture of one’s skin can provide information about their age, health, ethnicity, and beauty. Important! Researchers examined images and videos to have a better understanding of the skin (Usman Akram et al., 2013). Binary classification is used in image processing to find skin regions (Soille, 2003). Skin areas were identified *via* image processing; skin detection required training, preprocessing, and postprocessing. Skin detection uses colour pixels, light, and an acquisition device. Computation, occlusion equations, and the capacity to solve challenging issues are all required for skin detection. Light and reflection are two aspects that contribute to nonlinear skin detection because the illumination of most pixels cannot be detected during the preprocessing step, so an estimating approach is necessary. The static skin detection image preprocessing can assist in overcoming some of the challenges that emerge in the real world. Skin detection is a challenging academic subject to tackle (Sharma et al., 2020). These articles discuss several methods for improving the functionality of electrical devices. For algorithm-based skin cancer detection, you need reliable equipment. Figure 2 shows the different types of melanomas.

The sections that follow are as follows: In Section 2, the related approaches are explained, and Section 3 and Section 4 explain the proposed method and results, respectively. Section 5 contains the conclusion and summarises the findings and approaches.

## 2 Related work

In pre-processing and segmentation, thresholding, edge-based analysis, and region-based analysis are employed (Kashyap, 2018; Kashyap, 2021; Nair and Bhagat, 2021; Nair et al., 2021; Kashyap, 2022; Sharma et al., 2022). This chapter goes through the concept of threshold segmentation using clustering, global, and adaptive thresholding simultaneously. This treatment is successful when the lesion and the skin surrounding it can be identified. Abbas et al. (Abbas et al., 2011) performed automatic segmentation using double thresholding (Argenziano et al., 2002; Gonzalez and Woods, 2002; Celebi et al., 2008); describe lesion segmentation using edges. Gonzalez and Woods (Gonzalez and Woods, 2002) examined lesions using Laplacian-of-Gaussian zero-crossings as a segmentation method. Parameters that can change (Argenziano et al., 2002; Celebi et al., 2008) Active contouring Edge-based treatments are ineffective due to improper boundaries and little colour change in the treated zone. These issues can be overcome by employing active contouring with variable parameters. False borders can be caused by a variety of factors, including skin texture variances, colour reflections, and hair (Schmid, 1999). Morphological flooding (Argenziano et al., 2002), multi-resolution Markov random field expansion (Argenziano et al., 2002), and multiscale region expansion (Dinesh, 2007) are other approaches. Contrast and compare several dermoscopy segmentation methods; edge-focused techniques are not included in their comparisons. To find and split skin lesions, techniques such as thresholding, clustering, and grayscale area growth were developed. According to Abbas et al. (Abbas et al., 2011), both Lissner and Urban employ non-uniform colour spaces to boost DTAE outcomes (Lissner and Urban, 2012). For SRM and JSEG, spatially based active outlines are an effective method, and border recognition and image capture should be done in a clear and easy manner. During testing, select one image at random from a huge image database. To derive meaningful inferences from data, large samples are necessary; it is not recommended that the border recognition algorithm be trained using the test image collection. In an effective manner, share diagnostic images with others and select the strategy that puts the least amount of demand on the available resources. It is critical to compare the boundaries of the lesion with those of other dermatologists. You may be able to make greater use of the space and resources at your disposal if you employ boundary detection. Identifying skin cancer images will almost certainly need the use of a variational model (Gómez et al., 2008; Schaefer et al., 2011; Usman Akram and Khan, 2013; Emre Celebi et al., 2015). Mean-shift gradient vector flow has surpassed gradient vector flow and level setting as they have been done in the past. Using this cutting-edge technology, one may be able to find the most cost-effective energy-saving strategy. The method takes into account both the mass density and the gradient vector flow (Gonzalez et al., 2007). Both mean drift estimates and numerical optimization of support functions were crucial. In the deep learning process, images, audio, and text are all used to teach the system key qualities. Using a series of “layers”, deep learning can handle data that doesn’t follow a straight-line, pull-out features, and classify data (Gómez et al., 2008).

**FIGURE 3:**
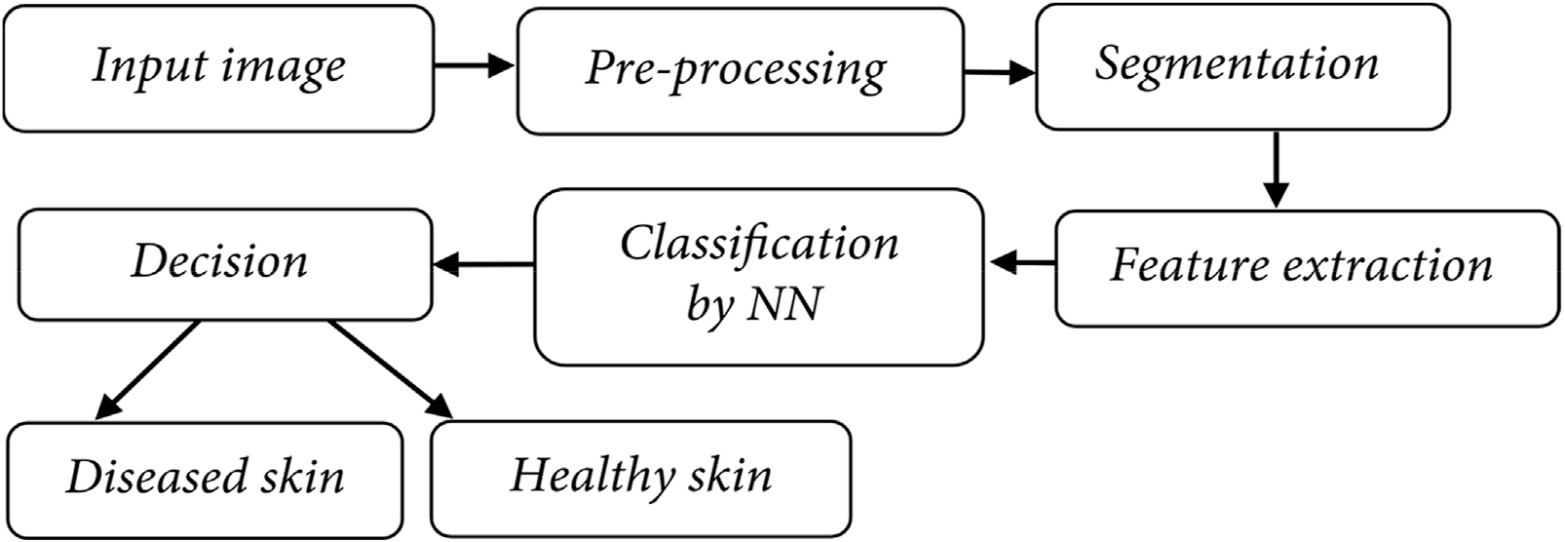
Skin cancer classification model.

The skin is the biggest organ in the body, yet it is also the most vulnerable. The skin is a typical site for sickness to appear. The purpose of this study is to develop, propose, and refine an algorithm for classifying skin conditions. The inclusion of many phases leads to more precise categorization. Figure 3 displays the fundamental phases, which are the same as those of existing systems.

This study illustrates how machine learning may be used to identify and categorise skin diseases, and it also recommends a better technique that could benefit human health. The most common types of skin cancer are melanoma, basal cell carcinomas, and squamous cell carcinomas. Melanoma is the most lethal form of the disease. The study also analyses and discusses the improved strategy. Skin detection requires both a strong algorithm and the most recent advances in computer technology. Machine learning makes use of preprocessing, segmentation, and feature extraction to deliver intelligent diagnosis (Argenziano et al., 2002). Preprocessing is the act of preparing data. To begin, “preprocessing” improves segmentation and reduces noise in a photograph. Both image noise and segmentation may be improved using preprocessing. This must be completed before processing can begin; the black frame itself, as well as air bubbles, skin lines, hairs, and blood vessels, can all cause noise. This study advises that modern approaches be used in the detection of skin cancers and in reducing radiation exposure from medical equipment. Deep learning approaches are used in the provided technique, which is acceptable.

Convolutional neural networks have also demonstrated considerable success in a wide range of pattern-finding applications. This was proven by the excellent work they produced. Dermatoscopy and colour photographs taken on a regular basis can be used to classify melanomas and other types of skin cancer. This is one method that can be used. To initialize the models, a variety of distinct pre-trained CNN modes are used, and there are many of them. VGG16, Densenet121, Xception, InceptionV3, EfficentNetB0, ResNet50V2, and Custom are a few examples. This is done so that the knowledge gained from studying one model can be applied to a different model’s understanding. In the first step of CNN, a model is trained using the training dataset and an appropriate number of iterations. In the second stage, it is determined which examples perform poorly considering the classification’s results. The goal is to use that data to iteratively train the next model. Once everyone involved has gotten the necessary instructions, the procedures can be carried out in an iterative fashion. We will then put the outcomes of the first two processes to the test to compare the overall performances of different models (Kumar and Vatsa, 2022). We will conduct this review when the first two stages have been completed. Finally, the problem of making predictions about samples is addressed using high-performing models that have been trained.

Furthermore, the procedure under consideration was carried out using the RNN approach. Because it uses feed-forward networks to carry out its functions, it behaves similarly to the human brain. RNNs can create more exact output predictions when applied to data sequences because they can recall crucial details of the data they are given. RNNs can now process larger volumes of data thanks to this capacity. Because input can only be transmitted forward through the network, this type of neural network is also known as a feed-forward-based neural network. The network’s name reflects the fact that it can only handle the data traveling section. The process begins with the layer designated for input, proceeds through the layers designated for intermediate processing, and finally terminates at the output layer, which is referred to as the final layer. Furthermore, it reacts to the inputs it is now receiving, and the only reason it can recall what happened in the past is because of the training it has received. RNN also employs a loop to cycle over the data iteratively (Jan et al., 2022). This is performed by iterating through the data. The outputs are collected, copied, and sent back into the network to be processed again before a decision is made on what to do with them. This happens before a decision is reached about what to do with them. The most recent data, as well as the lessons learned from previous inputs, are used to inform the conclusions it draws and the final judgments it takes. When making a choice, it is reasonable to believe that RNN analyses both the current situation and the recent past. Given the data, we can make this assumption with confidence.

## 3 Proposed method

This section explains how to distinguish between melanocytic and nevus lesions by eliminating hair, gel, uneven contrast, and other artifacts. Melanocytomas and nevi are classified, and hair identification, inpainting, changing the colour space, contrast stretching and augmentation and lesion segmentation are all procedures that have received approval. Dermoscopy is an essential component of the system; it removes important artefacts such as hairs so that the image may be improved and segmented more efficiently. Individual strands should be identified and highlighted before being inpainted. If you wish to get rid of the unwanted hair on your body, you must repent. Image enhancement allows you to distinguish between healthy and pathological skin. The final phase of the therapy is to remove any skin lesions that may have been present on the body.

### 3.1 Hair artifacts removal

After removing the hair artefacts, we go over the various strategies for segmenting the lesions that are accessible. Using this method, extra information about hair is given without categorising the hair.This is done by combining Gabor wavelet-based directional filters and augmentation with an inpainting segmentation technique based on neighbourhood estimation. These are used to extract the features of a similar structure group. To reduce the false-positive effect that hair has on the lesion segmentation process, the permitted hair artefact reduction strategy can be used. The removal of hair artefacts from a picture begins and a binary mask is applied to the picture to identify each individual hair inside it (Naseri et al., 2018a; Farouk et al., 2020). Using edge detection techniques and filters are used to remove hair, gel and uneven contrast. It is required to shave the affected region, replacing lost hairs across the neighborhood. Hairs are painted before lesion enhancement and segmentation may take place; this prevents the possibility of highlighted artefacts appearing after operations are completed.

### 3.2 Hair inpainting

**FIGURE 4:**
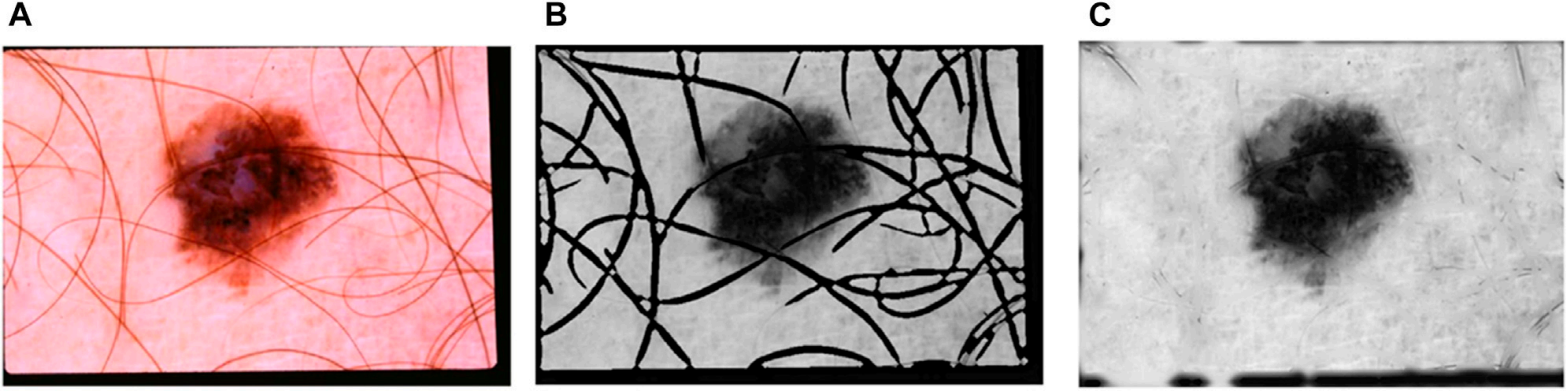
**(A)** An image with hair **(B)** A black and white image **(C)** image after hair inpainting operation.

To distinguish individual strands, a binary mask is used; to create a smooth image, the pixel for each strand must be filled in. The technique of precisely filling in binary hair masks using neighbourhood-based area filling (NBRF) is known as “hair inpainting”, and it can be seen extending outward from the object’s centre in all directions. Because background estimates and averages are used and because there is a smooth transition between each hair, the hairs in the filled area become more consistent each time they are drawn. Another obvious sign of ageing is a gradual slowing of the rate at which new hair grows in. Because it is a direct outcome of ageing, this is a clear indication that the body is ageing. This is a symptom like the body’s age-related decrease in the size of hair follicles. As the latter phases of the hair production cycle approach, the hair begins to partially emerge but splits from the base of the hair follicle. Your hair is always in the latent catagen phase to some degree, somewhere between 5 and 10% of the time. To fill in each pixel that represents a hair, cyclic morphology methods are used. Figure 4 depicts an enlarged view of the in-paint NBRF hairs.

In this part, the proposed method emphasises the vibrancy of the colour space (L). The brightness of the shot changes, and the lighting alters the contrast between the skin and any lesions.

**FIGURE 5:**
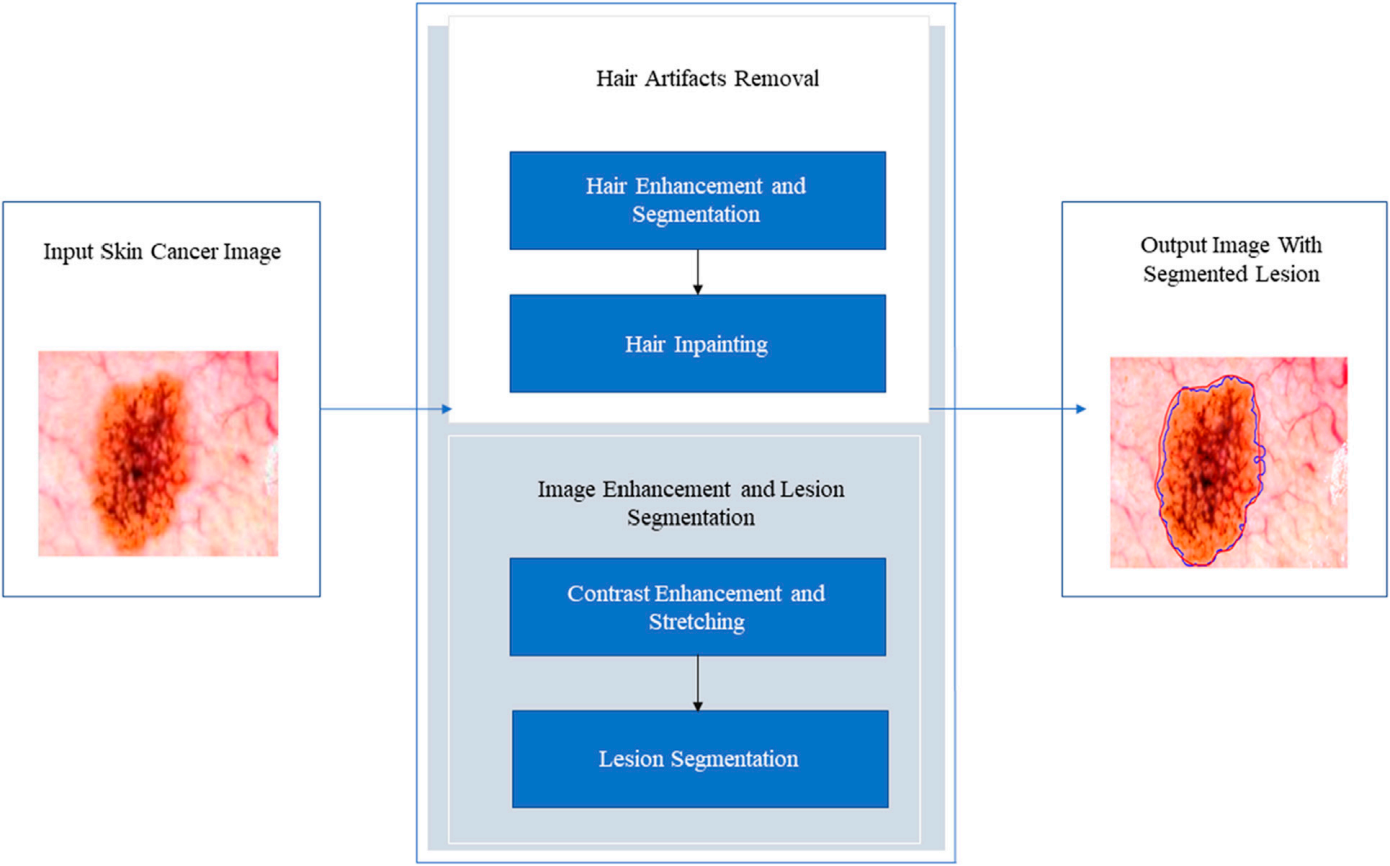
The proposed segmentation and classification process.

The pixel contrasts of the skin and lesions may differ significantly from one dermoscopy to the next. Equalizing the brightness of skin and lesion pixels while increasing contrast can aid in the successful segmentation of lesions. With the use of the recommended technology, we hope to be able to construct a computer vision detection system for segmenting and identifying skin problems. This approach would segment a skin lesion and identify the ailment based on image features. Figure 5 depicts an overall view of the proposed system.

Known-results training is required for machine learning or deep learning systems, and then, using early data, test your forecasts (extracted from a given image). Digital skin photographs frequently have bubbles and noise (Emre Celebi et al., 2015). Noise impedes segmentation and reduces feature quality, resulting in incorrect classification and prediction. This is corrected through image processing during segmentation; digital noise blurred the images. Utilizing noise-cancellation technology, N-training pictures are averaged by averaging eight still picture pixels in the vertical, horizontal, and diagonal directions (Schaefer et al., 2011). The proposed segmentation method is hampered by extreme visual inhomogeneity. Although its performance declines as inhomogeneity increases, the proposed method performs well for segmenting inhomogeneous images. The size of the model may only be altered once per picture because real-world inhomogeneity is unexpected, and the model should not rely on a single scale parameter. The proposed model aids in the management of inhomogeneous pictures; it is based on binary fitting and incorporates multiscale information, which may help to explain the overall visual properties. The model inherits the ability to segment inhomogeneous images from local features and disregards the original contours and Gaussian noise. When using the same scale on inhomogeneous images, the results will be erroneous.

### 3.3 Regulating

**FIGURE 6:**
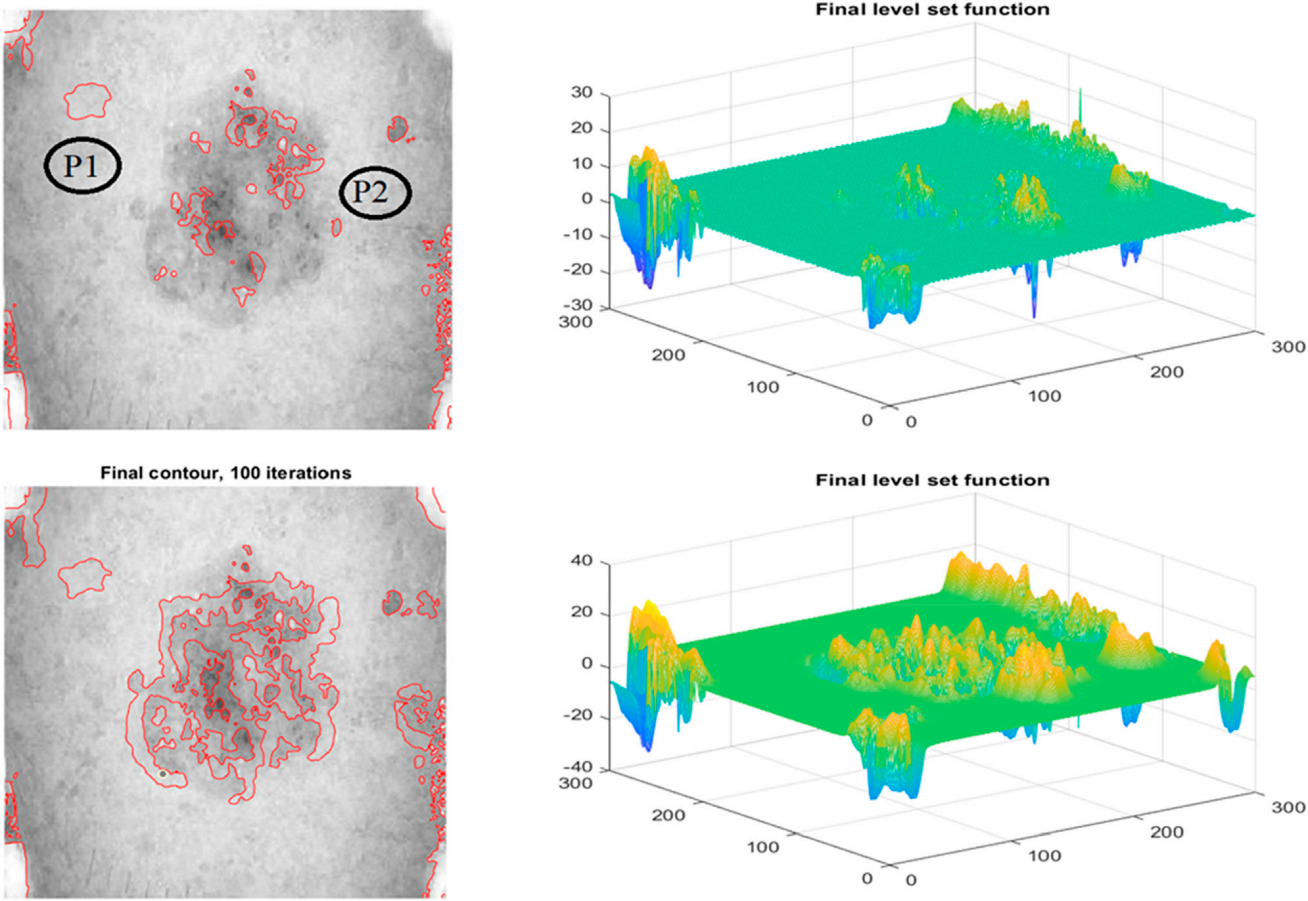
Row 1: The Proposed model Initial Segmentation and Level set plot Row 2: The Segmented Result and the respective level set function.

The improved level set function (LSF) is flat or approaches 0 during the LSE development. Consistent reinitialization allows the degraded LSF to operate as an SDF. Several computational methods for re-initializing LSF have been attempted, as shown in Figure 6. Even if comparable techniques have previously worked, it is nonetheless concerning. Don’t overlook the inner boundary to avoid mistakes during image segmentation. The operations of creating new zero contours and reinitialization consume CPU time and slow down LSE. The LSE is hampered by two issues. To regularise the LSF during development, variational level set formulations (Naseri et al., 2018b; Heidari et al., 2019; Krishnamoorthi et al., 2022) were created. Therefore, reinitialization may not be required to resolve previous issues. Zhang et al.'s method removes reinitialization and has a strong theoretical foundation. Each active contour iteration adds a certain amount of time to the previous one. Time step size constraints and speed convergence This problem is caused by the limitation of the signed distance function. The LSF gradient is not affected.

Figure 6 depicts the results of the proposed segmentation method. The first row depicts data heterogeneity: the currently selected pixel is shown by an x, and its 15-pixel neighborhood is circled. The image depicts two markers. There are two distributions: 41–64 and 157–181. Both distributions have grayscale spans of around 13, indicating that picture inhomogeneity changes little when image data is communicated from top to bottom. The second row of Figure 6 depicts the final segmented images. The number of iterations required to acquire the final goal contour and the CPU time required to complete segmentation is two methods for measuring how quickly the proposed model converges. Both are detailed further below. Our study contrasts it with three-level set models designed for inhomogeneous pictures. LBF, LGDF, and LIF are all examples of related types. The beginning curves and input images are comparable to Figure 6. Using these two strategies, iterations may be reduced from hundreds to dozens, and CPU time can be reduced from dozens of seconds to hundreds of milliseconds. Images show the texture and colour of the skin; this type of colour characteristic is commonly used for segmentation, and the development of specialised characteristics makes categorization difficult (Ahmad et al., 2021; Almarzouki et al., 2021). Texture is a feature of local intensity that may be used to identify impacted locations. The accuracy of classifiers is improved when frequency and space information are added to texture features.

**FIGURE 7:**
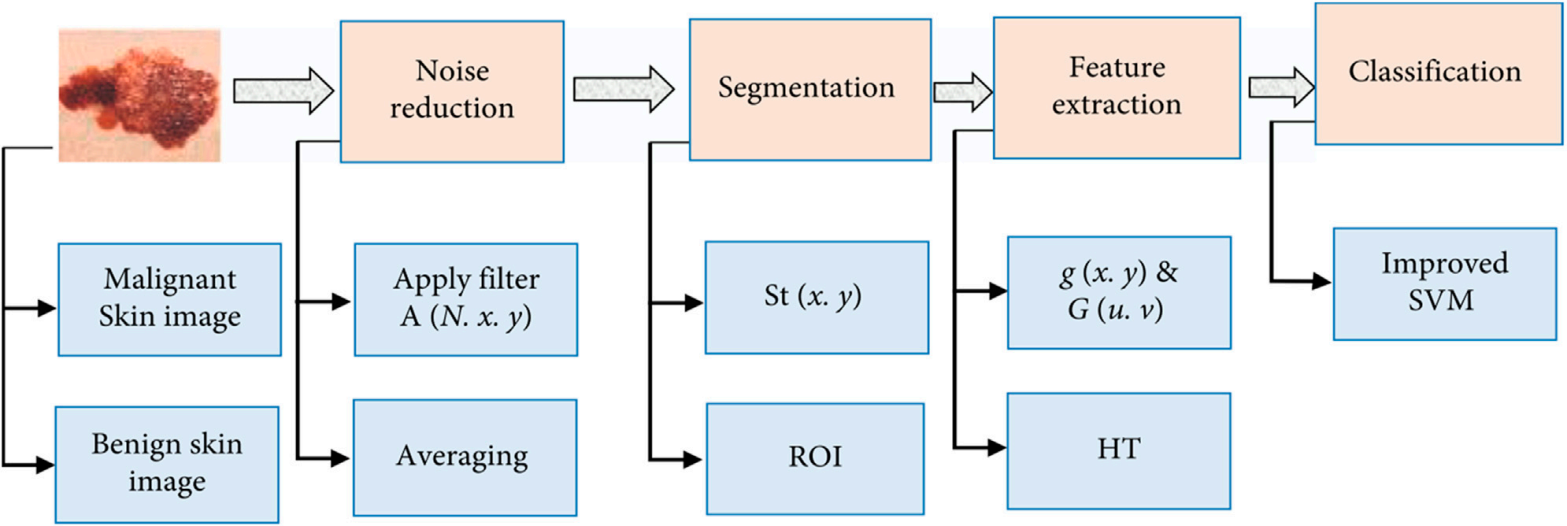
Processes within different stages of the proposed method.

Classification is necessary since it necessitates the application of judgement at every level. An SVM’s performance can be improved by first gaining control over it and then adapting it to the results desired by the algorithm (Ahmad et al., 2021; Anand et al., 2022a; Anand et al., 2022b; Anand et al., 2022c; Anand et al., 2022d; Hossen et al., 2022; Krishnamoorthi et al., 2022). At each moment in time, feature vectors can undergo dynamic transitions between x-activities. The weight of one activity differs from the weight of the other activities according to its weight. The proposed method may incorporate elements such as tumour clustering and illumination in certain locations shown in Figure 7. There is little inhomogeneity in this LBF picture segmentation sample. The grey histogram in the centre. (b) and (c) a histogram of grayscale levels. The histogram illustrates the many shades of grey that surround the centre point. Segmentation that is ineffective. A threshold separates a noise-free image, and this straightforward, accurate method effectively segments photos. Regions and links are used in segmentation. Images are converted to black and white by selecting a threshold (T) and separating pixels into equivalent sections. If a pixel has an intensity of T or above, it is an object; otherwise, it is the background. Segmentation is required for image processing and ROI (Gonzalez et al., 2007). Edge detection, region identification, and pixel classification are all part of segmentation. Clustering, feature extraction, and colour range segmentation are all possible.

Convolutional neural networks have been shown to be extremely effective in a wide range of pattern-finding applications. The high quality of their output demonstrates this. Dermatoscopy and colour photographs taken on a regular basis are beneficial for classifying melanomas and other skin malignancies. This strategy can be used in a variety of situations, and this is one of them. There are numerous pre-trained CNN modes, and they are all used during model initialization. VGG16, Densenet121, Xception, InceptionV3, EfficentNetB0, ResNet50V2, and Custom are some popular examples of such networks. This is done so that the lessons learned from studying the older model can be applied to the newer one more efficiently. The first stage of CNN involves training a model with the training dataset and an appropriate number of iterations. The classification results are then used in the second phase to identify the cases with the poorest performance. The goal is to use that data to iteratively train the next model. Once everyone has received the instructions they need, the steps can be done repeatedly.

**FIGURE 8:**
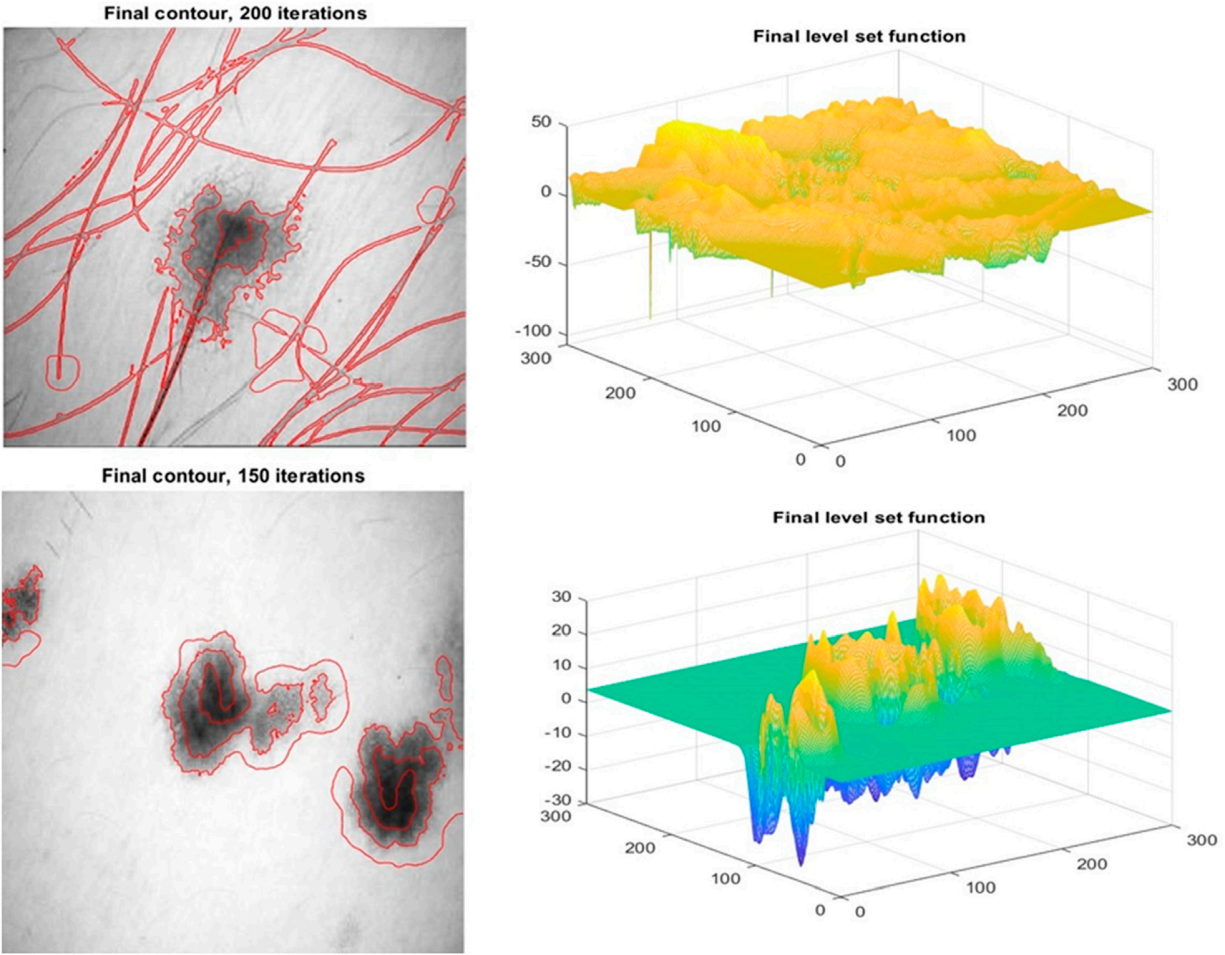
Minor feature of the cancerous cell along with the level set function drawings.

The image backdrop influences categorization, this method has been well researched. The extracted item is compared to the background using feature extraction. Figure 8 depicts cancer cell characteristics and level set function drawings. Non-linear data is any information that doesn’t fit neatly into one of a few categories that have already been set up. In situations like these, a non-linear support vector machine (SVM) classifier is used. Non-linear support vector machines are used when data analysis is needed, but it is hard to pick out a linear axis. This is because data needs to be properly separated along a linear axis for linear support vector machines to work. The employment of kernel functions allows the samples to be projected onto a high-dimensional feature space. This is done to make linear classification easier to perform. If the decision boundary is not linear, the SVM is likely to struggle with correctly classifying the data. Take a look at these examples; none of them provide any sign of a logical progression from one class to the next. In the support vector machine (SVM) technique, it is not possible to get non-linear decision boundary models from any direct theory.

## 4 Results

**FIGURE 9:**
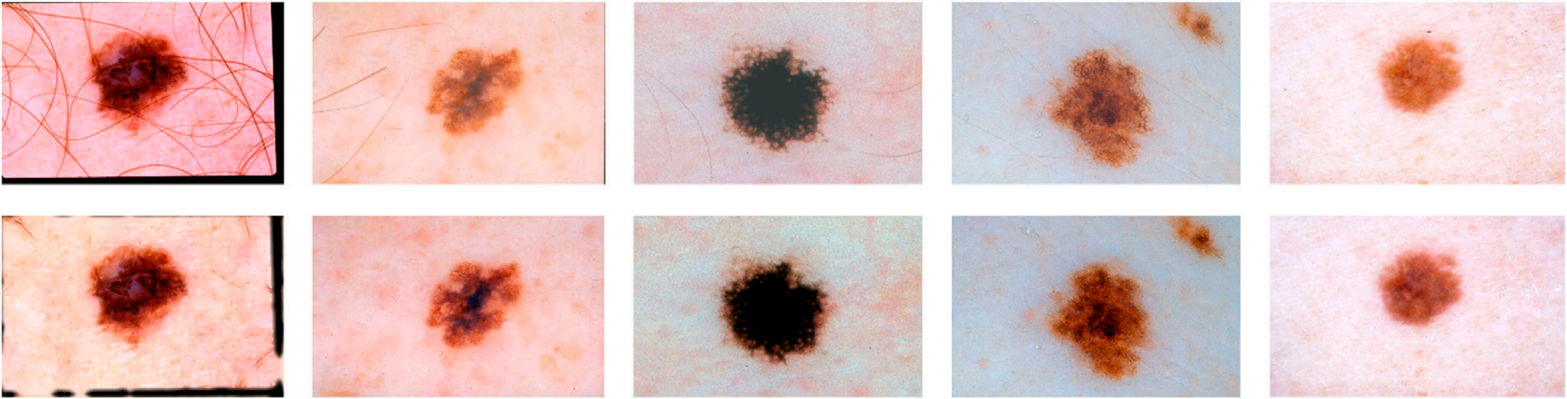
Skin cancer images after removing hair.

TPR, FPR, Jaccard Similarity, and Dice Similarity Coefficients are examples of measurements that may be used to compare the proposed approach against its rivals. In this experiment, we will test the efficacy of our method for eradicating hair artifacts. The segmentation is improving as filtered hair pixels are converted to skin pixels within the context of the suggested approach. Keeping hair pixels at bay Figure 9 demonstrates how to identify and localise hair artefacts in dermoscopy pictures. Images with varied hair densities were chosen to demonstrate how well the proposed technique works in real-world situations, even in the presence of artefacts. Figure 9 demonstrates that our approach is capable of precisely recognising each individual hair in the picture.

**FIGURE 10:**
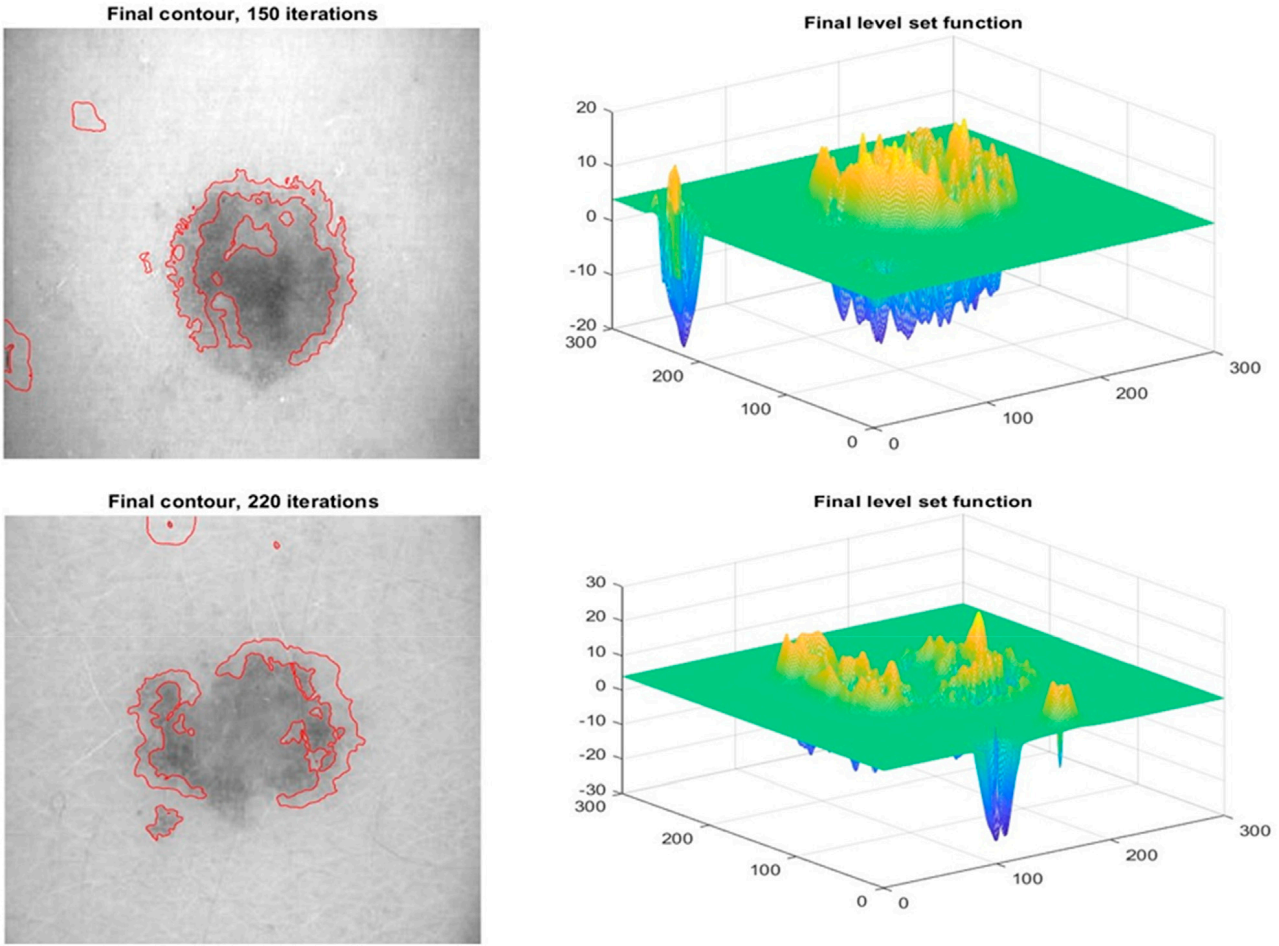
Small Features Detection using the proposed method.

This section examines the efficiency of contrast enhancement and stretching. This part includes lesion segmentation, a comparison to the ground truth, and the calculation of performance measures. They appear regularly in numerous works of literature. The MBD, JSeg, DTEA, and RAC techniques were examined and compared to the authorised lesion segmentation methodology. There are numerous ways to segment lesions that can be employed; we compared many segmentation algorithms. Table 2 shows the results of the segmentation done on dermoscopic images, as well as the approach’s performance. Comparisons are imaginable. The approach offered can obtain a true detection rate of 97.26 per cent. When compared to the three other approaches presently used to identify melanoma boundaries, the proposed method performs much better. To reliably distinguish between the skin and lesion pixels, the suggested technique eliminates hair aberrations and employs dynamic contrast and stretching. Figure 8 demonstrates the efficiency of segmentation. The segmentation performed manually by a dermatologist is shown in red, whereas the segmentation performed automatically by the system is shown in blue. The sample shows how the recommended approach may segment hairs, veins, gel, and varying levels of brightness and contrast. There is either too much or too little segmentation. This is caused by the colour and intensity of the lesion. The adaptive sigmoidal function accomplishes this aim by minimising the number of pixels impacted by the multitone lesion. These pixels indicate skin or artefacts that were not eliminated by the pre-treatment. Figure 8 depicts the extent to which the therapy was successful. Levelset function is shown in Figure 10.

The method was examined in this study using a typical image of tumour skin. If you have many images in your dataset and use a high-resolution image like the one you have here, your training system will function optimally. Early detection of cancer is critical to averting its possibly fatal effects. These technologies make early cancer detection simpler. They reduce the amount of effort required by specialists and provide trustworthy information regarding the progress of the illness, even if the diagnosis is made too late. Melanoma cells are notoriously dangerous to one’s health. In Table 1, you can see both the confusion matrix and how often it happens.

**TABLE 1:** The confusion matrix represents the proposed method’s recognition rate.

basal	93	6	5
squamous	3	89	4
melanoma	2	5	91
	basal	squamous	melanoma

The image depicts MBD in comparison to the real world. Both the border and the proposed technique are comparable. As a result, we ran parallel benchmarks on both our approach and the most recent methods, using the same system with the same hardware requirements as we would in real life. This enabled us to compare the effectiveness of both treatments. JS and DSC must be close to one, whereas FPR and FNR must be close to zero. Table 2 displays the FPR, FNR, DS, and Jaccard similarities. This action is carried out on each image using a global cut-off value. The distinction between healthy and unhealthy skin is striking. It is possible that the settings for another photo will not work.

**TABLE 2:** The Comparison of the various method with the indexes Jaccard Similarity, Dice Similarity Coefficient, False Positive Rate, and False Negative Rate.

Image	Method	Indexes			
		Jaccard similarity	Dice similarity coefficient	False positive rate	False negative rate
**Image 1**	Chan Vese	0.5806	0.7499	0.0560	0.6299
	Geodesic Active Contour	0.8222	0.9422	0.2293	0.3243
	EM	0.6202	0.7867	0.2280	0.5639
	OTSU	0.8252	0.9443	0.2963	0.2474
	PCNN	0.8243	0.9437	0.3099	0.2300
	The Proposed Method	**0.9403**	**0.9749**	**0.0492**	**0.0454**
**Image 2**	Chan Vese	0.5929	0.7604	0.0258	0.6258
	Geodesic Active Contour	0.4690	0.6394	0.0999	0.7374
	EM	0.9097	0.9959	0.2528	0.0766
	OTSU	0.0962	0.2847	0.0229	0.9059
	PCNN	0.2074	0.2933	0.0328	0.9946
	The Proposed Method	**0.9429**	**0.9093**	**0.0249**	**0.0649**
**Image 3**	Chan Vese	0.9046	0.9594	0.0564	0.0670
	Geodesic Active Contour	0.7499	0.8897	0.2344	0.3993
	EM	0.9035	0.9598	0.0392	0.0835
	OTSU	0.9537	0.9257	0.2400	0.0470
	PCNN	0.9900	0.9473	0.0960	0.0527
	The Proposed Method	**0.9402**	**0.9749**	**0.0238**	**0.0397**
**Image 4**	Chan Vese	0.8534	0.9632	0.0504	0.3449
	Geodesic Active Contour	0.6989	0.8506	0.0949	0.4850
	EM	0.8399	0.9542	0.0798	0.3392
	OTSU	0.8893	0.9864	0.2229	0.2482
	PCNN	0.8899	0.9868	0.2480	0.2224
	The Proposed Method	**0.9098**	**0.9959**	**0.0443**	**0.0496**
**Image 5**	Chan Vese	0.5645	0.7349	0.0555	0.6479
	Geodesic Active Contour	0.7974	0.9250	0.2049	0.3656
	EM	0.4599	0.6283	0.7609	0.0032
	OTSU	0.0860	0.2496	0.0208	0.9359
	PCNN	0.0850	0.2489	0.0269	0.9369
	The Proposed Method	**0.9440**	**0.9099**	**0.0204**	**0.0030**
**Image 6**	Chan Vese	0.9798	0.9404	0.0299	0.2258
	Geodesic Active Contour	0.8459	0.9582	0.0003	0.3762
	EM	0.9808	0.9409	0.0359	0.2096
	OTSU	0.9934	0.9542	0.0486	0.0867
	PCNN	0.9839	0.9432	0.0522	0.0942
	The Proposed Method	**0.9006**	**0.9588**	**0.0002**	**0.0244**

These bold values are the results of the proposed Method.

## 5 Conclusion

We provide a novel approach for segmenting dermoscopy images by using melanocytic and nevus lesions as dividing lines. Directed wavelet filters can be used to remove artefacts by increasing the number of pixels that make up hair and vessels. In this study, a level-set segmentation-based proposed model that segments inhomogeneous images is introduced. LSF may be proven without reinitialization by including reactive diffusion energy; this enables LSF testing without the need for reinitialization. The model in this study has excellent accuracy, speed, convergence, resilience to the contour start location, and noise interference. In the future, we hope to employ a probabilistic graphical model and a few more principles to improve the adaptability of our algorithm when segmenting real-world photos. When inpainting hairs and vessel pixels, NBRF considers the information around them. Uneven brightness can be reduced by adopting tactics such as luminance equalisation and non-uniform lighting, among others. The sigmoidal function aids in highlighting variances in the appearance of skin lesions. The image is enlarged, the threshold is decreased, and any other morphological operations that are required are carried out so that the lesion may be separated from the skin around it. We examined one hundred distinct dermoscopy photos, and each one revealed either benign lesions, nevi, or metastatic melanoma. The proposed technique yields findings with a true detection rate of 94.4 percent, a false positive rate of 3.62 percent, and an error rate of 3.39 percent. The testing results back up the claim that the recommended method for dividing up lesions is one that works well and is not affected by things like hairs, blood vessels, changing brightness, and contrasts.

## Data Availability

The original contributions presented in the study are included in the article/Supplementary Material, further inquiries can be directed to the corresponding author.
